# Perception of lean construction implementation barriers in the indian prefabrication sector

**DOI:** 10.1016/j.heliyon.2024.e36458

**Published:** 2024-08-18

**Authors:** Prafful Negi, Gaurav Thakur, Rajesh Singh, Anita Gehlot, Amit Kumar Thakur, Lovi Raj Gupta, Neeraj Priyadarshi, Bhekisipho Twala

**Affiliations:** a Department of Civil Engineering, Uttaranchal Institute of Technology, Uttaranchal University, Dehradun, 248007, India; b Division of Research & Innovation, Uttaranchal Institute of Technology, Uttaranchal University, Dehradun, 248007, India; c Lovely Professional University, Phagwara, Jalandhar, Punjab, 144001, India; d Department of Electrical Engineering, JIS College of Engineering, Kolkata, 741235, India; e Digital Transformation Portfolio, Tshwane University of Technology, Staatsartillerie Rd, Pretoria West, Pretoria, 0183, South Africa

**Keywords:** Construction sector, India, Lean construction, Prefabrication, Sustainable construction, Waste reduction

## Abstract

Prefabricated construction, increasingly recognized as a sustainable method, enhances productivity while mitigating the drawbacks of traditional approaches. Lean construction, pivotal for sustainability, targets waste reduction and cost efficiency while delivering value to customers. In India's prefabrication sector, numerous barriers impede the implementation of lean principles, necessitating their identification and resolution to advance lean practices. This study aims to identify and analyze primary barriers to implementing lean principles within India's prefabrication industry, focusing on professionals' perceptions. Employing exploratory factor analysis, it examines these barriers' interconnections and causal relationships, providing actionable recommendations for enhanced lean construction effectiveness. Through a review of the literature, 26 significant barriers were identified and primary data was obtained with the help of a questionnaire. 25 barriers were discerned after pre-exploratory factor analysis, culminating in ten common components. Notably, the study highlights a primary barrier: understanding of lean construction. Drawing from expert insights, substantial recommendations are provided, intending to guide the prefabricated building sector in overcoming barriers to on-site lean construction. These findings and recommendations offer valuable direction for industry stakeholders.

## 1 Introduction

The construction industry in India holds a significant position in the economy, directly contributing to the Gross Domestic Product (GDP) and providing a significant number of jobs to the populace [1]. One of the offshoots of the construction industry is the prefabrication construction industry in which building components or modules are manufactured in a controlled environment off-site, then transported and assembled on-site to create a complete structure. Prefabrication offers a variety of benefits, including improved energy efficiency, waste reduction, effective construction, quality, safety, quick execution, and sustainability. Reducing construction time also lowers labor costs and enables quick principal return [2].

Prefabrication, however, does not just have advantages; it also faces several challenges. The prefabrication method necessitates skilled workers to operate modern machinery and equipment for production, transportation, and assembly [3,4]. However, due to the novelty of this approach, many workers lack experience, prompting companies to conduct training sessions before commencing construction [5,6]. Consequently, this incurs additional costs, and experienced workers command higher salaries than their inexperienced counterparts for the same roles [7,8]. Manufacturers' delays in supplying components when needed, the absence of necessary machinery and transportation facilities for component transfer, workers' lack of prefabrication expertise, high initial construction costs, zero margin for error during component manufacturing, and shorter lifespans are significant challenges encountered in prefabrication construction [9,10]. To address these challenges, lean principles are being implemented within the prefabrication industry.

Lean principles, derived from the Toyota Production System, are aimed at minimizing waste and maximizing value delivery [11]. These principles include identifying value, streamlining processes, creating flow, establishing pull, and continuously improving processes to enhance efficiency and quality. Lean construction applies these principles specifically to the construction industry, focusing on eliminating waste, improving project flow, reducing lead times, enhancing collaboration, and maximizing value for all stakeholders involved in construction projects [[12], [13], [14]]. Lean Construction involves adopting lean management techniques and tools, serving as a production management-based approach to completing projects by ensuring that production, supply, and assembly of products occur on schedule and in the required quantity [15]. Emphasizing the balance in the utilization of people, materials, and assets, Lean Construction works to reduce expenditure, eliminate waste, and accelerate project delivery duration [16].

The United Nations (UN) established the UN 2030 Agenda in 2015 to achieve Sustainable Development Goals, aiming to safeguard everyone's well-being while preserving, restoring, and encouraging the sustainable use of ecosystems. Both sustainability and Lean Construction share common objectives, including promoting resource efficiency and minimizing waste, which directly contribute to the attainment of SDG Goal 11 (Sustainable Cities and Communities) [17]. Additionally, prefabrication supports the achievement of SDG goal 9 (innovation, industry & infrastructure) and goal 11 (sustainable cities & communities).

Lean construction has been implemented in the Indian prefabrication construction industry to eliminate waste, improve project flow, and deliver projects more efficiently and cost-effectively [[18], [19], [20], [21], [22]]. Prefabrication construction has become increasingly popular in India, with many companies now applying lean construction methodology to leverage its benefits. However, despite its adoption by many prefabrication firms to address low profitability and enhance competitiveness, lean construction encounters several barriers when applied to the prefabrication industry. These barriers include challenges related to supply chain management, lack of senior-level support, multilayer subcontracting, lack of lean construction training and education, worker expertise, initial construction costs, and resistance to change [[23], [24], [25], [26], [27]].

Despite platforms like the International Group for Lean Construction (IGLC) facilitating global collaboration and knowledge exchange among construction professionals, including those in India, various barriers are faced during the implementation of lean principles in the prefabrication construction sector in India. Identifying and addressing these barriers is essential for advancing lean practices in the Indian prefabrication construction sector. Thus, the study aims to identify and analyze the primary barriers faced during the implementation of lean principles within the prefabrication industry in India, with a focus on understanding the perception of these barriers among construction professionals. Additionally, it seeks to provide valuable insights into the interconnections and causal relationships among these barriers, thereby offering actionable recommendations to enhance the effectiveness of lean construction practices in the prefabrication sector.

The main objectives of the study are as follows.

• Identify and analyze primary barriers to implementing lean principles within India's prefabrication industry, focusing on professionals' perceptions.
• Employ exploratory factor analysis to examine interconnections and causal relationships among these barriers.
• Provide actionable recommendations for enhancing lean construction effectiveness based on the analysis.
• Guide the prefabricated building sector in overcoming barriers to on-site lean construction.

The research work is illustrated below: the relevant literature review is covered in Section 2; a methodological framework is proposed in Section 3. Section 4 demonstrates the exploratory factor analysis performed to determine each barrier's weight and recognize common components, and the obtained results are analyzed and discussed finally, Section 6 shows the conclusion of the study and some recommendations.

## 2 Review of literature

This section offers insights into Lean Construction (LC) and its advancements, prefabrication construction, and the relationship between LC and prefabrication, with a focus on developing countries. It also sheds light on India's specific circumstances, particularly within the prefabrication construction sector, emphasizing the importance of identifying critical barriers to lean implementation.

### 2.1 Lean construction & key advances

Lean Construction has significantly advanced the construction industry by enhancing efficiency and maximizing value throughout project lifecycles [28]. Initially championed by Glenn Ballard and influenced by Lauri Koskela, LC has evolved with several key innovations. Glenn Ballard is credited with developing the Last Planner System (LPS), which enhances project planning and coordination by involving stakeholders in collaborative planning processes [29]. This approach aims to improve predictability and reduce uncertainties in project timelines [30]. Lauri Koskela introduced the Theory of Flow Value (TFV), emphasizing the maximization of value and reduction of waste through streamlined production processes [31,32]. TFV utilizes methodologies such as Value Stream Mapping (VSM) and Root Cause Analysis (RCA) to systematically identify and eliminate non-value-adding activities [33].

Beyond these foundational contributions, LC has integrated practices like Integrated Project Delivery (IPD), which fosters collaboration among project stakeholders from early design stages through project completion [34]. IPD aligns incentives and goals among stakeholders, promoting a cooperative approach to project delivery and risk management. Additionally, LC principles have been applied to supply chain management, focusing on just-in-time delivery to reduce lead times, improve material flow, and minimize inventory costs and waste. Building Information Modeling (BIM) has also emerged as a pivotal technology within LC, leveraging digital tools to enhance collaboration, visualization, and decision-making throughout the construction process [35].

Furthermore, lean supply chain management techniques have streamlined material procurement, delivery, and inventory management, resulting in reduced waste and improved coordination [36]. The emphasis on continuous improvement within the lean construction culture encourages project teams to apply techniques like value stream mapping and root cause analysis, driving innovation and optimization throughout construction projects [37,38]. Other advancements include Just-In-Time (JIT), 5S, 6S, and Total Quality Management (TQM) are used to reduce waste, increase efficiency, and improve quality [39]. These advancements collectively signify a paradigm shift towards more collaborative, cost-effective, and sustainable approaches to completing building projects.

### 2.2 Prefabrication construction

Prefabrication, characterized by the assembly of building components off-site before transportation to the construction site, has garnered significant attention in the industry due to its potential to revolutionize traditional construction practices. One of the primary advancements in prefabrication lies in its ability to enhance construction efficiency and productivity through off-site fabrication, reducing on-site labor requirements and accelerating project schedules [[40], [41], [42]]. This approach also facilitates greater precision and quality control, minimizing errors and defects during assembly [43,44]. Moreover, prefabrication promotes sustainability by optimizing material usage, minimizing waste generation, and reducing the environmental impact associated with traditional construction methods [[44], [45], [46]]. However, despite these advancements, prefabrication presents several challenges that warrant critical consideration. One such challenge is logistical complexity, as coordinating the transportation and assembly of prefabricated components requires meticulous planning and coordination among various stakeholders [47,48]. Additionally, ensuring compatibility and integration between prefabricated elements and on-site construction activities demands robust communication and collaboration throughout the project lifecycle [49]. Furthermore, prefabrication may encounter resistance from traditional construction stakeholders hesitant to adopt innovative methodologies, necessitating comprehensive change management strategies to overcome cultural barriers and foster industry-wide acceptance [50]. Addressing these challenges requires a holistic approach that integrates technological innovation, strategic planning, and stakeholder engagement to unlock the full potential of prefabrication in construction [44].

### 2.3 Relation between lean construction and prefabrication

Prefabrication and lean construction support the same goals and share similarities such as simplifying the construction process, reducing waste, saving time and costs, and achieving sustainability. Integrating makes them compatible. When applied to prefabrication, LC principles offer significant potential to optimize production workflows, improve project efficiency, and enhance overall project outcomes [36,51]. One key area where LC enhances prefabrication is in streamlining production processes to minimize waste and increase productivity [52,53]. By adopting lean production techniques such as Just-in-Time (JIT) manufacturing and Kanban systems, prefabrication facilities can optimize material flow, reduce inventory, and minimize lead times, resulting in leaner, more efficient operations [27,54]. Furthermore, LC promotes collaborative planning and coordination among project stakeholders, facilitating seamless integration between prefabricated elements and on-site construction activities [53,55]. Through techniques like the Last Planner System (LPS) and collaborative scheduling, LC fosters communication, fosters early identification of potential bottlenecks, and enables proactive problem-solving to ensure project timelines are met [31,32]. Additionally, LC encourages a culture of continuous improvement, empowering prefabrication teams to regularly evaluate processes, identify areas for enhancement, and implement iterative changes to drive performance gains [56].

Another crucial aspect is the positive impact on health and safety (H&S). Prefabrication in a controlled factory environment reduces exposure to on-site hazards such as weather conditions, falls from height, and heavy machinery accidents [57]. LC's emphasis on streamlined workflows and clean, organized workspaces further enhances safety by minimizing risks associated with clutter and disorganization [58]. By integrating H&S considerations into lean practices, prefabrication not only improves efficiency but also creates safer working conditions for construction workers [58,59].

Despite these potential benefits, challenges exist in implementing LC principles within prefabrication contexts [36]. These challenges include resistance to change from entrenched organizational cultures, the need for specialized training and skill development among prefabrication workers, and the complexity of integrating lean methodologies into existing production systems [60]. Moreover, variations in project scope, design complexity, and client requirements can introduce additional complexities that must be navigated to realize the full potential of LC-enhanced prefabrication [61]. Numerous research endeavors are currently underway to explore the adaptability of lean concepts and methodologies to the realm of prefabrication construction, mirroring their utilization in conventional construction practices. These studies delve into diverse aspects such as enhancing quality standards [37], fostering lean education and training initiatives [36], optimizing resource utilization [62], and refining on-site scheduling procedures [62].

Prefabricated buildings are also part of the construction industry and share common attributes. Hence, research on barriers to LC implementation, which did not specifically target prefabricated buildings, was examined to provide valuable reference for this study, as illustrated in Table 1. However, given the cultural and economic differences among countries and the unique construction process of prefabricated buildings, it is essential to conduct an in-depth analysis of integrating LC with prefabricated buildings in the context of India.

**Table 1:** Barriers to the implementation of lean construction in the construction industry in different countries [[63], [64], [65], [66], [67], [68], [69], [70], [71], [72], [73], [74], [75], [76], [77], [78], [79], [80], [81]].

**S. No.**	**Barriers**	**Countries**
1.	Insufficient Awareness	United Kingdom, India, Bangladesh, Malaysia, Chile, Palestine, Saudi Arabia, Morocco
2.	Resistance to change	United States, China, United Kingdom, Bangladesh, Malaysia, Palestine, South Korea, South America, Pakistan, Morocco
3.	Insufficient support from senior leadership	United Kingdom, Bangladesh, Palestine, China, Pakistan, Malaysia, Morocco
4.	Challenges with cooperation	Bangladesh, United Kingdom, Palestine, Pakistan, Finland, Libya, Uganda
5.	Lack of relevant incentives	United Kingdom, Bangladesh, Pakistan, Palestine
6.	Market competition hindering the adoption of lean construction practices	United Kingdom, Bangladesh, China, Morocco
7.	Absence of performance evaluation	Palestine, Bangladesh, Libya, Saudi Arabia
8.	Unskilled workforce	Pakistan, Uganda, Morocco, India
9.	Inadequate training and education on lean construction	Chile, Bangladesh, Pakistan, United Kingdom, South Korea, United States, China, South America, Brazil, Saudi Arabia, Singapore, Palestine
10.	Lack of suitable lean technology or tools	Bangladesh, United Kingdom, Palestine, United States, South Korea, South America, China, Pakistan, Saudi Arabia, Libya, Uganda
11.	Insufficient funding during construction	Bangladesh, Palestine, United Kingdom, Morocco, Pakistan
12.	Complex subcontracting layers	Morocco, China, Bangladesh
13.	Lack of organizational structure and culture for lean construction	Chile, Bangladesh, United Kingdom, United States, South America, South Korea, Pakistan, Saudi Arabia, China, Uganda, Morocco, Libya
14.	Ineffective supervision and control	United Kingdom, Morocco
15.	Insufficient standardization	Bangladesh, United Kingdom, Saudi Arabia, Finland, Uganda
16.	Limited personal empowerment	United Kingdom, South America, United States, South Korea, Chine
17.	Poor program planning	United Kingdom, Uganda, Bangladesh, Morocco

### 2.4 Lean construction in developing countries

Developing regions, particularly in Asia and Africa, confront more extensive challenges when implementing lean construction practices [81]. The prospects of lean implementation are significantly influenced by economic and technological development [81]. In recent years, many developing countries have initiated lean initiatives to reduce waste and enhance project value [25]. However, effective lean adoption requires a robust framework that considers the economic, environmental, social, political, and cultural aspects specific to each developing nation [49]. China, as the largest developing country, encounters various barriers to implementing lean construction in the prefabrication sector, such as inadequate education and training on lean construction, a lack of sufficient lean tools or technologies, and insufficient support from senior leaders [82]. Additionally, challenges include resistance to changes in lean construction practices and a lack of organizational culture and structure regarding lean construction [83]. Similarly, Bangladesh faces barriers such as a lack of understanding of lean construction [66], cooperation issues, intense market competitiveness leading to a limited window for adopting new technologies [84], and inadequate performance reviews [85].

In India, the initiation of Lean construction practices found its impetus through the establishment of the Institute of Lean Construction Excellence (ILCE) in 2008. This non-profit organization represents a collaborative effort among seven prominent construction firms in India, committed to embracing Lean principles. Functioning as a frontline in the Lean movement, ILCE operates in conjunction with esteemed academic institutions like the Indian Institute of Technology-Madras (IITM), leveraging their expertise as knowledge partners. Despite the progress made in preparing for Lean adoption, India, like many developing nations, also encounters barriers to implementing lean construction in the prefabrication construction sector [31,86,87]. Table 2 presents the identified barriers to implementing lean principles in the prefabrication construction sector.

**Table 2:** Identified barriers to implementing lean principles in the prefabrication construction sector.

**S. No.**	**Barriers**	**Source**
1.	Understanding of lean construction	[44,46,80]
2.	Resistance to adopting better tools & techniques	[42,45,49]
3.	Lack of motivation due to the absence of relevant incentives	[47,48,50]
4.	Unskilled workers	[29,31,32]
5.	Insufficient program planning (ineffective scheduling and sequencing of materials, equipment, and labor)	[39,40,52]
6.	Lack of coordination among different departments	[40,43,46]
7.	Difficulty in collaboration between teams due to multilayer subcontracting	[31,34,76]
8.	Market competition makes it difficult to adopt lean construction practices	[41,44,46]
9.	Inadequate professional management capabilities of managers	[60,64,69]
10.	Lack of effective supervision and control	[78,79]
11.	High turnover of the workforce	[41,42,48]
12.	Lack of support from persons at senior levels	[34,44,48]
13.	Insufficient standardization of prefabrication processes	[25,35,65]
14.	Poor quality safety training	[41,45,69]
15.	Insufficient funds and excessive cost savings during construction	[24,34,69,80]
16.	Lack of coordination outside the construction department	[67,77,80,81]
17.	Complex projects and highly uncertain environment	[64,80,86]
18.	Waste due to double handling, over-communication, re-communication, and additional quality assurance checks/inspection	[15,64,69]
19.	Limited training opportunities for new workers about tools, equipment, and techniques	[69,78,81]
20.	Construction workers are left idle on a site, leading to inflated labor expenditure	[[83], [84], [85]]
21.	A mistake during the manufacturing of prefabricated components	[15,37,69]
22.	The complexity of prefabrication works	[15,69,80]
23.	Inefficient supply chain management	[68,78,83]
24.	Inaccurate documentation	[79,83]
25.	Wasted talent due to excessive firm organizational hierarchies	[64,80]
26.	Over-estimating, over-ordering, or the untimely procurement of inventory	[46,68,86]

This highlights the urgent need to identify and address the “Critical Barriers to Lean Implementation in the Prefabrication Construction Industry” within the Indian context. Consequently, the main objective of this study is to elucidate the barriers to implementing Lean construction methodologies within the prefabrication construction sector in India and to provide actionable insights to promote sustainable Lean implementation practices in the region.

## 3 Research methodology

This section provides an overview and outlines the research methods utilized for the study, employing a structured questionnaire survey approach for data collection and analysis. In the following subsections, the research methodology is delineated, detailing the rationale behind the chosen approach, the steps undertaken in data collection and analysis, and the ethical considerations guiding the research process.

### 3.1 Research design

**Fig. 1:**
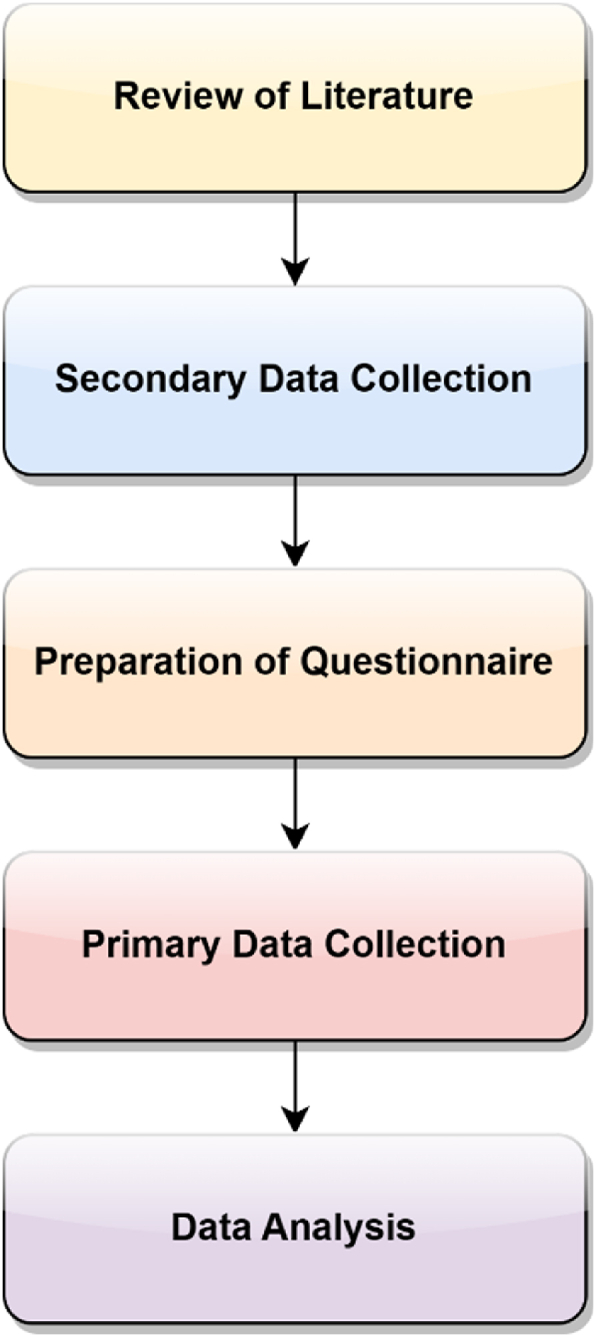
Research approach.

The structured questionnaire survey method was employed for data collection due to its effectiveness in systematically gathering data from a diverse sample of respondents in a standardized manner, ensuring consistency and comparability of responses. This approach allows for the collection of specific information relevant to the research objectives and provides a framework for organizing data, facilitating analysis, and interpretation [86]. Moreover, it offers a practical means of reaching a large and geographically dispersed population, enabling the study to capture a comprehensive range of perspectives and insights [87]. Fig. 1 illustrates the research methodology employed in the study.

### 3.2 Data collection

The initial phase involved collecting secondary data, which entailed identifying significant barriers to implementing lean construction in the prefabrication industry through an extensive review of the literature. To identify gaps and fulfil the study objectives, the Systematic Literature Review (SLR) method was employed, allowing for systematic and comprehensive coverage of literature within specific time duration. A total of 26 barriers to the implementation of lean construction in the prefabrication construction sector were identified, as depicted in Table 2. The common barriers were then ranked based on their frequency of occurrence and their perceived significance within the domain of lean construction in the prefabrication industry. This approach provided a strong foundation for the subsequent stages of our research, including the questionnaire design and data analysis.

After identifying significant barriers, a structured questionnaire was prepared. To enhance the reliability and robustness of the survey instrument, the questionnaire design process involved referencing established measurement scales and validated instruments. The questionnaire utilized a Likert scale ranging from 1 to 5 to gauge the impact level, with 1 representing “Strongly Disagree”, 2 denoting “Disagree”, 3 indicating “Uncertain”, 4 signifying “Agree”, and 5 representing “Strongly Agree”. The questionnaire underwent rigorous validation procedures, including the Kaiser-Meyer-Olkin (KMO) and Bartlett's test to ensure its accuracy, reliability, and validity. Validity describes how well the measured results match the questions that are being asked. The higher the validity, the more closely the measured result resembles the subject matter under examination. The KMO test is used to investigate partial correlation and inter-variable correlation. The value of the KMO is utilized to confirm that the factor analysis can be employed for a certain collection of data. Formula 1 is used to show partial correlation and correlation among the variables.

(1) $$KMO=\frac{X\left(a_{i},a_{j}\right)}{X\left(a_{i},a_{j}\right)+Y\left(a_{i},a_{j}\right)}$$

X (a_i_,a_j_) = The sum of the squares of the correlation coefficients across all variables.

Y (a_i_,a_j_) = The sum of the squares of the partial coefficient of correlation among all variables.

Limits of the values are KMO>0.9 (Excellent), KMO>0.8 (Good), KMO>0.7 (Acceptable), KMO> 0.6 (Questionable), and KMO<0.5 (Unacceptable) [78].

A pilot survey was also conducted before the formal questionnaire to assess its effectiveness, clarity, and reliability. This pilot survey involved a small group of individuals representing the target audience. The purpose was to identify any issues, ambiguities, or biases in the questionnaire. Feedback from the pilot survey was used to refine and improve the questionnaire, ensuring it was clear, user-friendly, and capable of generating reliable responses in the final survey. The pilot survey helped enhance the overall quality of data collected in the main study. The finalized questionnaire was then distributed to target respondents via Google Forms through social media platforms and emails. This aimed to collect perceptions of professionals regarding barriers to implementing lean construction in the prefabrication construction sector (primary data) for further analysis.

### 3.3 Sampling

Regarding participant selection, a purposive sampling technique was employed to target professionals working in the Indian prefabrication sector. This sampling method allowed us to select participants who possessed relevant expertise and experience in the subject matter [87]. The participants were identified based on specific criteria such as their roles, experience levels, and involvement in lean construction practices, to ensure representation from various sectors within the industry. To achieve this, a structured approach was taken.

a) **Criteria Definition:** Participants needed at least three years of construction experience, including one year in prefabrication or lean construction, ensuring they had the expertise to provide valuable insights.
b) **Targeted Outreach:** Industry professionals from various organizations, including construction firms, consultancies, academic institutions, and industry associations, were targeted. Invitations were sent to individuals who held positions such as engineers, contractors, manufacturers, consultants, surveyors, cost consultants, architects, developers, territorial heads, project managers, and other managerial professionals, ensuring a broad spectrum of perspectives.
c) **Verification and Inclusion:** Participants meeting the predefined criteria, as mentioned above, were recruited via professional networks, industry events, and online platforms. This approach ensured a knowledgeable sample directly involved with the subject matter. Efforts were made to include participants from diverse geographic regions across India for a comprehensive perspective capture.

As for the sample size, a total of 150 participants were included in the study. This sample size was determined using a 5 % confidence level sample estimation formula from Cochran, taking into account the population size and desired confidence level. The chosen sample size was deemed sufficient to achieve the study objectives and provide statistically reliable results. The response rate for the study was calculated to be 72.22 %. The primary data collected from the structured questionnaire survey was then utilized statistically to identify barriers to the implementation of Lean Construction for effective prefabricated project delivery in the Indian construction sector.

Ethical standards were rigorously upheld, ensuring participant confidentiality and data privacy. All respondents provided informed consent, and the study followed guidelines set by institutional review boards and regulatory bodies to maintain research integrity. A conceptual framework, as discussed in sub-section 3.4, was developed, and the identified barriers were tested and measured using the statistical tool SPSS.

### 3.4 Data analysis

The reliability and accuracy of the collected data were assessed using Cronbach's Alpha test, which provides insights into the consistency and dependability of data obtained through a structured questionnaire survey. This test calculates Cronbach's alpha coefficient (Formula 2), denoted by ‘α’, with values ranging between zero and one. A Cronbach's alpha coefficient value above 0.5 is considered suitable for analysis, indicating that the collected data is reliable [86].

(2) $$\alpha =\frac{y\left[1-\left(\sum_{i=1}^{y}\sigma^{2}x_{i}\right)/\sigma_{x}^{2}\right]}{y-1}$$

After ensuring the reliability of the collected data, the analysis employed the factor analysis approach, including both pre-exploratory and formal exploratory factor analyses, conducted using IBM SPSS (Statistical Package for the Social Sciences) software. Exploratory Factor Analysis, a classical technique utilized to condense a large number of variables and reduce dimensions, was applied. This technique aims to identify interdependent relationships among variables, grouping them into a smaller number of causal latent variables with independent relationships [83]. Pre-exploratory factor analysis was initially conducted to preprocess the gathered data and eliminate insignificant barriers.

Following this, Principal Component Analysis (PCA) was employed, serving as a component extraction technique. PCA aimed to transform the dataset with correlated variables into a smaller set of uncorrelated variables, known as principal components. Its objective was to minimize information loss while capturing maximum variance from the original data. Communality, representing the percentage of variance in each observed variable accounted for by the extracted main components, was calculated. This aided in evaluating overall data structure and patterns, facilitating the selection of pertinent variables for the research. Subsequently, after dimension reduction via common component extraction, classification was conducted, and detailed statistical analysis was performed to determine the weight of each barrier. Through this process, the top five barriers were identified, informing the formulation of recommendations to address and resolve these challenges.

### 3.5 Expert consultations

To address the identified top five challenges, recommendations were formulated through expert engagement, which involved structured discussions and brainstorming sessions facilitated by the research team. These sessions aimed to explore potential solutions by leveraging the expertise and best practices of the experts. Expert consultations involved professionals with extensive experience in prefabrication and lean construction, covering various aspects of the construction industry such as project management, process optimization, safety, quality control, and workforce management. The composition of the expert group aimed to ensure a comprehensive and multidisciplinary perspective on the barriers and potential solutions. This diverse group aims to provide comprehensive solutions. The process adheres to a systematic and collaborative approach, detailed below, for developing substantive recommendations.

a) **Identifying Top Barriers:** The initial step entailed identifying the five most critical barriers, based on their impact on lean construction implementation in the prefabrication sector.
b) **Expert Involvement:** The research team engaged with a panel of construction industry experts renowned for their profound knowledge. These experts offered invaluable insights into overcoming the identified barriers.
c) **Structured Discussions:** Expert engagement included structured discussions and brainstorming sessions, guided by the research team. These sessions focused on exploring potential solutions, drawing from the experts' knowledge and best practices.
d) **Prioritizing Recommendations:** Recommendations were methodically developed in order of priority, addressing the most crucial solutions first. The expert group's input played a pivotal role in determining each recommendation's priority.
e) **Cross-Validation:** Recommendations underwent thorough cross-validation through consensus-building with the expert group. This step ensured that the proposed solutions were practical, actionable, and aligned with industry best practices.
f) **Finalizing Substantive Recommendations:** The research team synthesized the recommendations based on expert insights. These recommendations were then structured and presented cohesively, as outlined in the study.

## 4 Result analysis

The findings of the analysis of the data gathered are covered in this section.

### 4.1 Statistics of the respondents

**Fig. 2:**
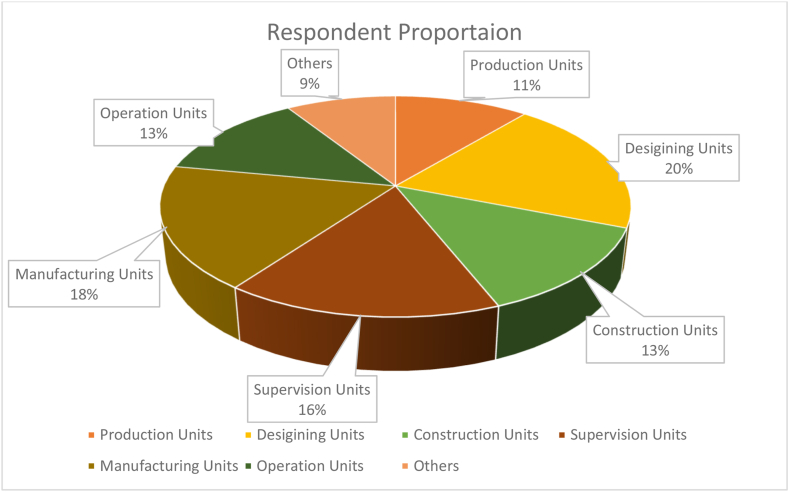
Distribution of respondent's work units.

**Fig. 3:**
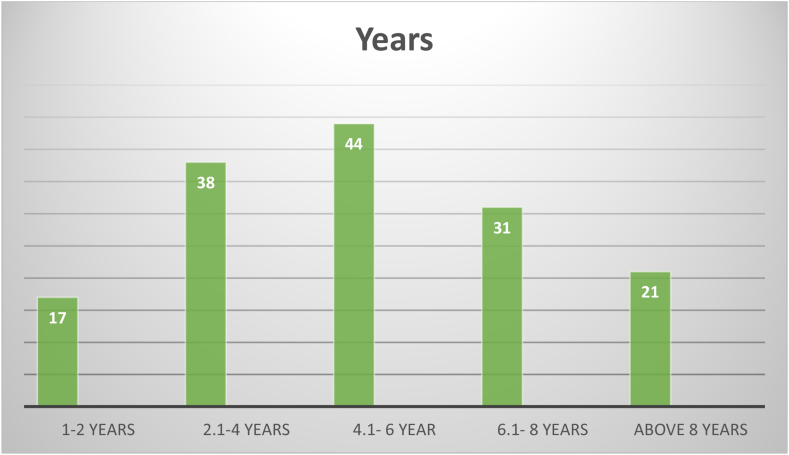
Experience of the respondents.

Between March 5 and April 26, 2023, a total of 151 questionnaire responses were collected, surpassing the threshold of 150, which is a fundamental requirement for conducting exploratory factor analysis [85]. The distribution of respondents' work units is depicted in Fig. 2, while Fig. 3 illustrates the years of experience respondents have accrued in the prefabrication sector. The majority of participants boast experience ranging from 4.1 to 6 years, followed by those with 2.1–4 years of experience, then individuals with 6.1–8 years of experience, and finally those with over 8 years of experience. These statistics affirm that respondents are highly qualified and have accumulated substantial experience over the years, ensuring the reliability and validity of the data collected for the study.

Fig. 2, Fig. 3 accurately depict the diverse range of experience levels within the industry, ensuring that the survey captures a broad spectrum of perspectives. The sample size for each category was determined based on the prevalence of professionals within these experience brackets, thus reflecting the actual composition of the workforce in the prefabrication sector. It's crucial to note that in survey research, the sample size should mirror the distribution of the population to yield results that are representative and applicable beyond the surveyed sample. Therefore, the selection of the sample aligns with the real-world distribution of experience levels among respondents, offering a comprehensive and realistic portrayal of the industry.

### 4.2 Validity & reliability of the questionnaire

Using SPSS, the validity of the questionnaire was assessed, yielding a Kaiser-Meyer-Olkin (KMO) value of 0.543, with a significance value of 0.000, as detailed in Table 3. Although falling within the questionable range, this KMO value did not dissuade the study from proceeding with Exploratory Factor Analysis (EFA). While it does not meet the criteria for excellent, good, or acceptable sampling adequacy, it hovers on the border of what is typically considered unsuitable for EFA. Typically, a KMO value below 0.5 is deemed unacceptable for EFA; however, in this case, despite being in the questionable range, it permitted meaningful insights into the factors influencing lean construction implementation in the prefabrication industry to be gleaned. The decision to proceed with EFA was based on specific research objectives, the nature of the data, and the necessity to identify factors influencing lean construction implementation in prefabrication. Despite interpreting the results cautiously, this study employed robust statistical techniques and theoretical justifications to ensure validity.

**Table 3:** Result of KMO & Bartlett's tests for validity analysis of questionnaires.

**KMO and Bartlett's Test**		
Kaiser-Meyer-Olkin Measure of Sampling Adequacy		0.543
Bartlett's Test of Sphericity	Approx. Chi-Square	1264.971
	Df	325
	Sig.	0.000

Cronbach's alpha coefficient was obtained as 0.679 as shown in Table 4 which is a parameter for good reliability of the collected data.

**Table 4:** Result of Cronbach's Alpha for reliability of questionnaires.

**Reliability Statistics**		
Cronbach's Alpha	Cronbach's Alpha Based on Standardized Items	N of Items
0.679	0.678	26

### 4.3 Results of exploratory factor analysis

After identifying a total of twenty-six key barriers, pre-exploratory factor analysis was employed to eliminate any insignificant barriers from the dataset. Principal Component Analysis (PCA) was then conducted to determine communalities and identify any unimportant barriers. Initially, each component's communality is set to one, reflecting the total number of components being equal to the total number of the first observable component. However, components with an extraction communality of less than 0.5 are considered insignificant and are disregarded [88]. Consequently, one component, “Inaccurate documentation,” exhibited an extracted communality value below 0.5, prompting its deletion. As a result, the original number of components was reduced to twenty-five after data pre-processing. Table 5 illustrates the communalities results before and after data pre-processing.

**Table 5:** Communalities result.

**Factors**	**Communalities Before Data Pre-Processing**		**Communalities After Data Pre-Processing**	
	**Initial**	**Extraction**	**Initial**	**Extraction**
Understanding of lean construction	1.000	0.920	1.000	0.923
Resistance to adopting better tools & techniques	1.000	0.613	1.000	0.620
Lack of motivation due to the absence of relevant incentives	1.000	0.619	1.000	0.620
Unskilled workers	1.000	0.666	1.000	0.678
Insufficient program planning (ineffective scheduling and sequencing of materials, equipment, and labor)	1.000	0.585	1.000	0.606
Lack of coordination among different departments	1.000	0.690	1.000	0.692
Difficulty in collaboration between teams due to multilayer subcontracting	1.000	0.636	1.000	0.640
Market competition makes it difficult to adopt lean construction practices	1.000	0.680	1.000	0.683
Inadequate professional management capabilities of managers	1.000	0.633	1.000	0.619
Lack of effective supervision and control	1.000	0.740	1.000	0.740
High turnover of the workforce	1.000	0.573	1.000	0.618
Lack of support from persons at senior levels	1.000	0.705	1.000	0.700
Insufficient standardization of prefabrication processes	1.000	0.912	1.000	0.918
Poor quality safety training	1.000	0.770	1.000	0.774
Insufficient funds and excessive cost savings during construction	1.000	0.600	1.000	0.641
Lack of coordination outside the construction department	1.000	0.665	1.000	0.682
Complex projects and highly uncertain environment	1.000	0.699	1.000	0.697
Waste due to double handling, over-communication, re-communication, and additional quality assurance checks/inspection	1.000	0.692	1.000	0.688
Limited training opportunities for new workers about tools, equipment, and techniques	1.000	0.690	1.000	0.690
Construction workers are left idle on a site, leading to inflated labor expenditure	1.000	0.688	1.000	0.686
A mistake during the manufacturing of prefabricated components	1.000	0.833	1.000	0.847
The complexity of prefabrication works	1.000	0.695	1.000	0.699
Inefficient supply chain management	1.000	0.606	1.000	0.622
Inaccurate documentation	1.000	0.488	–	–
Wasted talent due to excessive firm organizational hierarchies	1.000	0.572	1.000	0.576
Over-estimating, over-ordering, or the untimely procurement of inventory	1.000	0.644	1.000	0.638

The weights and rankings of the twenty-five components were measured and presented in Table 6. Using the weightage of the collected responses, the barriers were ranked accordingly. In cases where barriers have the same weightage, comparisons were made based on the Likert Scale values, and subsequent rankings were assigned accordingly.

**Table 6:** Weightage and ranking of the barriers.

**S. No.**	**Factors/Barriers**	**Total No. of Responses for Each Likert Scale**					**Weightage (%)**	**Rank**
		**1**	**2**	**3**	**4**	**5**		
1.	Lack of coordination among different departments	7	29	0	69	46	7.08	1st
2.	Understanding of lean construction	7	28	0	71	45	6.92	2nd
3.	Poor quality safety training	8	30	2	72	39	6	3rd
4.	Limited training opportunities for new workers about tools, equipment, and techniques	7	34	0	71	39	6	4th
5.	Unskilled workers	5	39	4	64	39	6	5th
6.	High turnover of the workforce	17	35	2	63	34	5.23	6th
7.	Insufficient funds and excessive cost savings during construction	5	33	0	81	32	4.92	7th
8.	Construction workers are left idle on a site, leading to inflated labor expenditure	18	36	4	65	28	4.31	8th
9.	Lack of effective supervision and control	3	45	2	75	26	4	9th
10.	Difficulty in collaboration between teams due to multilayer subcontracting	9	41	3	72	26	4	10th
11.	Wasted talent due to excessive firm organizational hierarchies	9	50	5	62	25	3.85	11th
12.	Lack of motivation due to the absence of relevant incentives	14	50	0	63	24	3.69	12th
13.	A mistake during the manufacturing of prefabricated components	12	39	6	71	23	3.54	13th
14.	Inadequate professional management capabilities of managers	1	48	0	80	22	3.38	14th
15.	Lack of support from persons at senior levels	12	38	1	79	21	3.23	15th
16.	The complexity of prefabrication works	19	41	1	69	21	3.23	16th
17.	Insufficient standardization of prefabrication processes	9	59	5	57	21	3.23	17th
18.	Complex projects and highly uncertain environment	21	51	2	56	21	3.23	18th
19.	Waste due to double handling, over-communication, re-communication, and additional quality assurance checks/inspection	11	38	5	78	19	2.92	19th
20.	Resistance to adopting better tools & techniques	28	38	1	66	18	2.78	20th
21.	Market competition makes it difficult to adopt lean construction practices	28	38	1	66	18	2.77	21st
22.	Over-estimating, over-ordering, or the untimely procurement of inventory	9	43	4	78	17	2.61	22nd
23.	Inefficient supply chain management	12	52	0	69	16	2.46	23rd
24.	Insufficient program planning (ineffective scheduling and sequencing of materials, equipment, and labor)	9	51	6	70	15	2.31	24th
25.	Lack of coordination outside the construction department	8	56	4	68	15	2.31	25th

*Where 1 – Strongly Disagree, 2- Disagree, 3- Uncertain, 4- Agree, 5- Strongly Agree.

After data processing, the formal implementation of an exploratory factor analysis began. The communalities before and after data processing, as shown in Table 5, indicated that each variable's extracted communality was more than 0.5. As depicted in Table 7, Table 8, the KMO and Cronbach's Alpha values shifted to 0.531 and 0.672, respectively, with a significance level of 0.001.

**Table 7:** Result of KMO &Bartlett's tests for validity analysis of questionnaires.

**KMO and Bartlett's Test**		
Kaiser-Meyer-Olkin Measure of Sampling Adequacy		0.531
Bartlett's Test of Sphericity	Approx. Chi-Square	1223.819
	Df	300
	Sig.	0.000

**Table 8:** Result of Cronbach's Alpha for reliability analysis of the collected data.

**Reliability Statistics**		
Cronbach's Alpha	Cronbach's Alpha Based on Standardized Items	N of Items
0.672	0.671	25

Using Principal Component Analysis (PCA), similar components as well as all components were collated and extracted, as shown in Table 9. The variance of each component was its eigenvalue, representing the total of the square loadings of all its original observable variables. The percentage of variance for each component was calculated by dividing its eigenvalue by the total eigenvalues of all the other components. For the total of twenty-five components, their combined total variance was 25. If a component's eigenvalue was less than 1, it could only account for one of the original observable variables. Therefore, after the calculation, the ten components with eigenvalues greater than 1 were selected as the common components. The ten components listed in Table 9's “Rotation Sums of Square Loadings” column contributed to 69.185 % of the total variance explained, exceeding the 60 % threshold for satisfactory construct validity [89].

**Table 9:** Principal component analysis explaining total variance.

**Total Variance Explained**									
**Component**	**Initial Eigen Values**			**Extraction Sums of Squared Loadings**			**Rotation Sums of Squared Loadings**		
	**Total**	**% of Variance**	**Cumulative %**	**Total**	**% of Variance**	**Cumulative %**	**Total**	**% of Variance**	**Cumulative %**
1	3.102	12.409	12.409	3.102	12.409	12.409	2.370	9.479	9.479
2	2.252	9.009	21.419	2.252	9.009	21.419	2.033	8.132	17.611
3	2.128	8.512	29.930	2.128	8.512	29.930	1.840	7.358	24.969
4	2.046	8.183	38.113	2.046	8.183	38.113	1.730	6.920	31.889
5	1.539	6.158	44.271	1.539	6.158	44.271	1.713	6.852	38.741
6	1.419	5.678	49.949	1.419	5.678	49.949	1.649	6.595	45.335
7	1.373	5.490	55.439	1.373	5.490	55.439	1.648	6.590	51.926
8	1.284	5.135	60.574	1.284	5.135	60.574	1.523	6.092	58.018
9	1.121	4.484	65.058	1.121	4.484	65.058	1.470	5.881	63.899
10	1.032	4.127	69.185	1.032	4.127	69.185	1.321	5.286	69.185
11	0.895	3.581	72.766	–	–	–	–	–	–
12	0.850	3.400	76.166	–	–	–	–	–	–
13	0.768	3.070	79.237	–	–	–	–	–	–
14	0.725	2.900	82.137	–	–	–	–	–	–
15	0.655	2.622	84.758	–	–	–	–	–	–
16	0.608	2.434	87.192	–	–	–	–	–	–
17	0.572	2.288	89.480	–	–	–	–	–	–
18	0.538	2.153	91.632	–	–	–	–	–	–
19	0.451	1.803	93.436	–	–	–	–	–	–
20	0.399	1.595	95.031	–	–	–	–	–	–
21	0.374	1.497	96.528	–	–	–	–	–	–
22	0.322	1.290	97.818	–	–	–	–	–	–
23	0.292	1.169	98.986	–	–	–	–	–	–
24	0.236	0.946	99.932	–	–	–	–	–	–
25	0.017	0.068	100.000	–	–	–	–	–	–
Extraction Method: Principal Component Analysis.									

**Fig. 4:**
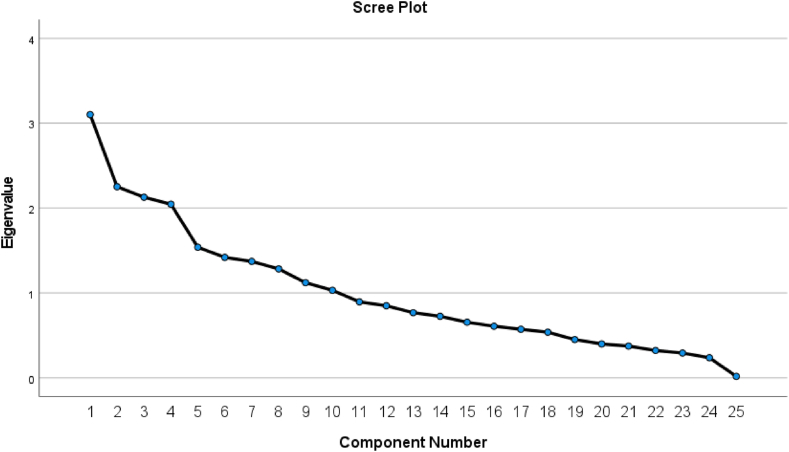
Screen plot.

For the evaluation of the logic of common component extraction, a screen plot was used. It appeared sensible to keep the first ten components as common components since the curve in Fig. 4 started to converge from the tenth component.

Through the varimax rotation approach, which changed the distribution of the percentage of variation explained by each component by rotating the axis, the original barriers were accordingly divided into the 10 common components. Because this technique simplified the component structure and maintained the cumulative proportion of the 10 common components, it was utilized for analysis. The rotated component matrix is shown in Table 10.

**Table 10:**
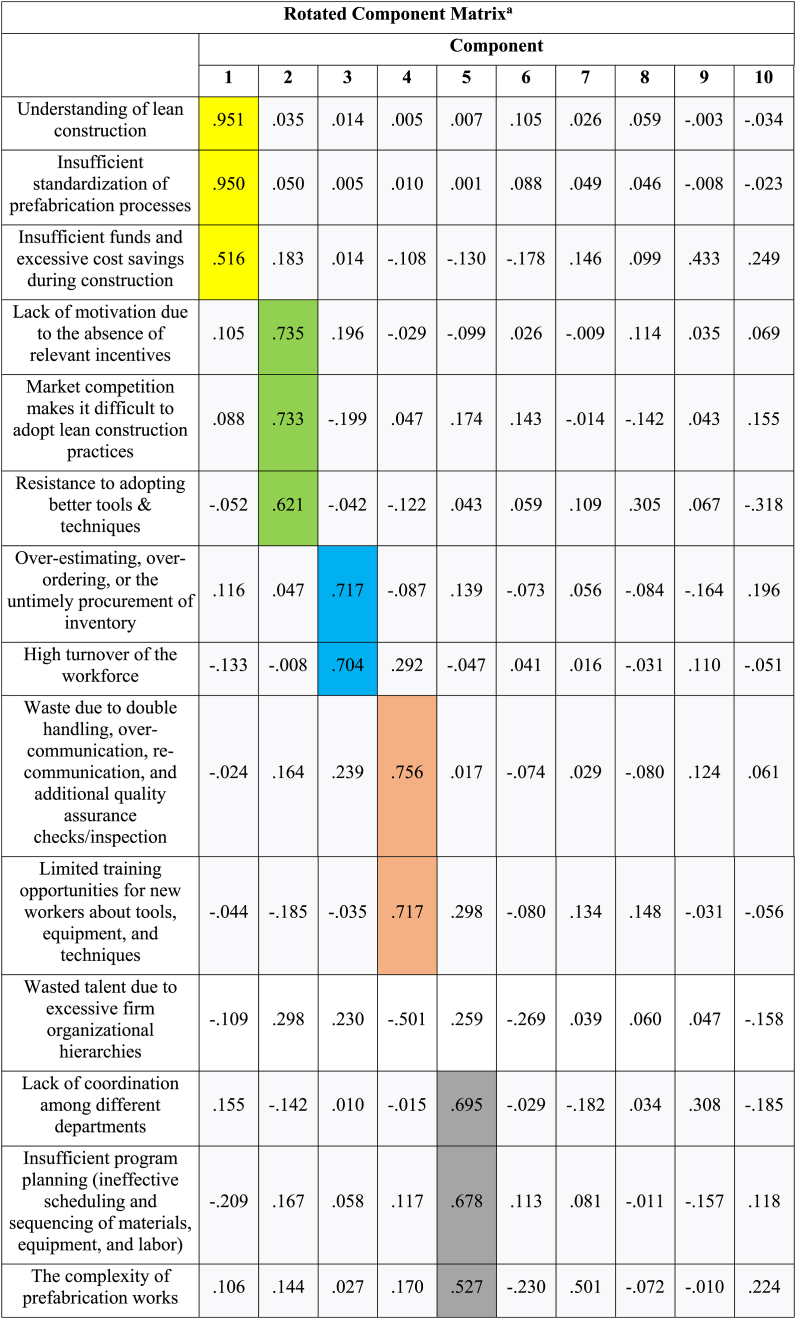

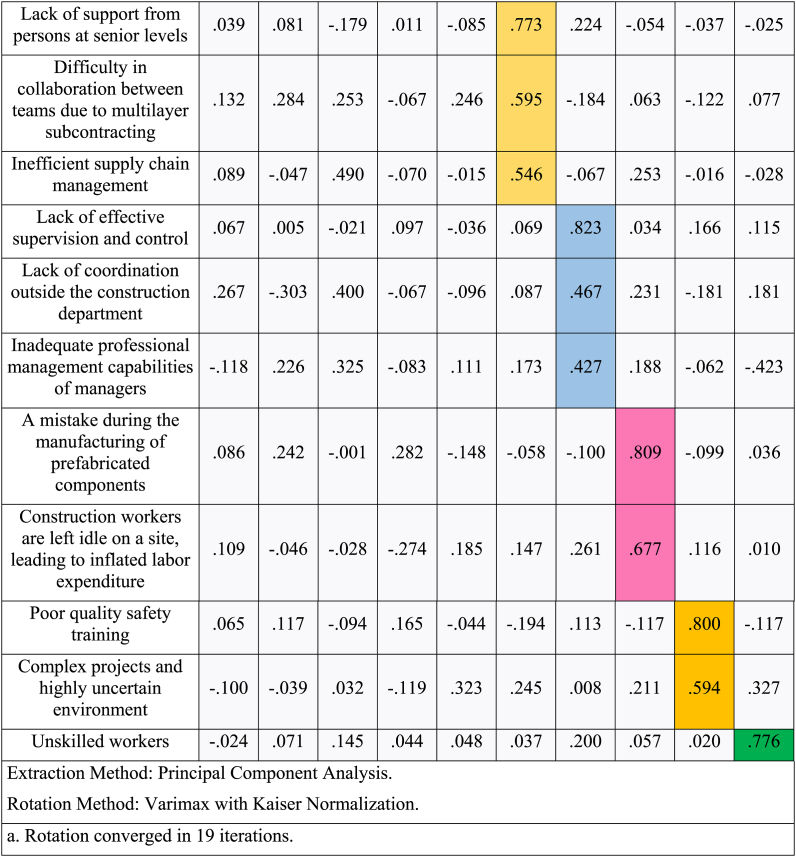
Rotated component matrix.

Following is an analysis of the common component extraction results.

• Component 1 consists of “Understanding of lean construction”, “Insufficient standardization of prefabrication processes” and “Insufficient fund and excessive cost saving during construction.”
• Component 2 consists of “Lack of motivation due to absence of relevant incentives”, “Market competition makes it difficult to adopt lean construction practices”, and “Resistance to adopting better tools & techniques.”
• Component 3 consists of “Over-estimating, over-ordering, or the untimely procurement of inventory”, and “High turnover of workforce.”
• Component 4 consists of “Waste due to double handling, over-communication, re-communication, and additional quality assurance checks/inspection” and “Limited training opportunities to new workers about tools, equipment, and techniques.”
• Component 5 consists of “Lack of coordination among different departments”, “Insufficient program planning (ineffective scheduling and sequencing of materials, equipment, and labor), and “Complexity of prefabrication works.”
• Component 6 consists of “Lack of support from persons at senior levels”, “Difficulty in collaboration between teams due to multilayer subcontracting, and “Inefficient supply chain management.”
• Component 7 consists of “Lack of effective supervision and control”, “Lack of coordination outside the construction department”, and “Inadequate professional management capabilities of manager.”
• Component 8 consists of “Mistake during manufacturing of prefabricated components”, and “Construction workers are left idle on a site, leading to inflated labor expenditure.”
• Component 9 consists of “Poor quality safety training”, and “Complex projects and highly uncertain environment.”
• Component 10 consists of “Unskilled workers.”

The research findings illustrate the favourable impact of lean construction principles within the prefabrication sector. However, substantial challenges hinder their effective implementation. The study has identified a total of twenty-five interconnected barriers, making it difficult to address them individually. Table 9, Table 10 show the results of the analysis and discussion, revealing the extraction of ten common components from the initial twenty-six barriers. These components are presented separately in Table 11, Table 12 for clarity, and the cumulative percentage reaches 68.984 %, exceeding the recognized threshold of 60 % for satisfactory conceptual validity.

**Table 11:** Percentage of variance of different components.

**Components**	**% of Variance**
Component 1	9.479
Component 2	8.132
Component 3	7.358
Component 4	6.920
Component 5	6.852
Component 6	6.595
Component 7	6.590
Component 8	6.092
Component 9	5.881
Component 10	5.286

**Table 12:** Common components and corresponding barriers in lean construction implementation

**Component**	**Name of the Component**	**Barriers**
Component 1	Understanding and Standardization	Understanding of lean construction
		Insufficient standardization of prefabrication processes
		Insufficient funds and excessive cost savings during construction
Component 2	Motivation and Adoption	Lack of motivation due to the absence of relevant incentives
		Market competition makes it difficult to adopt lean construction practices
		Resistance to adopting better tools & techniques
Component 3	Inventory and Workforce Management	Over-estimating, over-ordering, or the untimely procurement of inventory
		High turnover of the workforce
Component 4	Efficiency and Training	Waste due to double handling, over-communication, re-communication, and additional quality assurance checks/inspection
		Limited training opportunities for new workers about tools, equipment, and techniques
Component 5	Coordination and Complexity	Lack of coordination among different departments
		Insufficient program planning (ineffective scheduling and sequencing of materials, equipment, and labor)
		The complexity of prefabrication works
Component 6	Support and Collaboration	Lack of support from persons at senior levels
		Difficulty in collaboration between teams due to multilayer subcontracting
		Inefficient supply chain management
Component 7	Supervision and Management	Lack of effective supervision and control
		Lack of coordination outside the construction department
		Inadequate professional management capabilities of the manager
Component 8	Manufacturing and Labor	A mistake during the manufacturing of prefabricated components
		Construction workers are left idle on a site, leading to inflated labor expenditure
Component 9	Safety and Project Complexity	Poor quality safety training
		Complex projects and highly uncertain environment
Component 10	Workforce Skills	Unskilled workers

The identified and categorized 10 key components represent clusters of barriers that are impeding the implementation of lean construction in the Indian prefabrication industry.

### 4.4 Expert recommendations

The findings from expert consultations provide valuable insights into overcoming barriers to lean construction implementation within the prefabrication sector. This section outlines prioritized recommendations derived from expert consultations aimed at overcoming barriers to lean construction in the prefabrication sector. Following is the list of recommendations (listed in order of priority).

a) **Enhancing Departmental Coordination:** To improve coordination across various departments, it is imperative to delineate each individual's role in achieving the overarching objectives. Open and transparent communication must be promoted throughout different departments. Employees should gain insight into the operations and challenges of other departments to rectify errors effectively. The construction process should be dissected, clearly defining the responsibilities of each functional department. Stringent adherence to departmental deadlines is essential, and any encountered issues must be promptly communicated to other departments.
b) **Promoting Employee understanding:** Prefabrication companies should conduct regular employee and worker gatherings and seminars. These events should educate participants on new and advanced techniques and procedures and their impact on project outcomes. Implementing Lean Construction as a regular practice within prefabrication firms is crucial for increasing worker's understanding. Inserting Lean Construction terminology into contracts provides an effective means of enforcing these principles when necessary.
c) **Enhancing Safety understanding:** Workers should be well-informed about machinery, tools, and equipment to foster safety understanding. Proper training sessions should be conducted to educate workers on safe handling techniques, addressing emergencies and accidents, rendering first aid to victims, and observing necessary precautions such as wearing safety gear. Post-training evaluations should be carried out to assess the application of acquired knowledge. Valuable worker feedback should be obtained to continually improve safety measures.
d) **Providing Ongoing Training:** Regular training should be provided to familiarize workers with new tools, equipment, and practices. Knowledgeable and experienced individuals should be engaged to inform workers about contemporary construction techniques and their role in reducing project time, costs, and resource waste. These practices should also contribute to a safer work environment.
e) **Addressing Skills Development:** Prefabrication construction requires skilled workers due to its specific construction processes. Lean construction education and training are effective means to enhance workers' professional capabilities. Post-training evaluations are essential for determining individual skills and should be considered in decisions related to compensation and advancement opportunities.

**Fig. 5:**
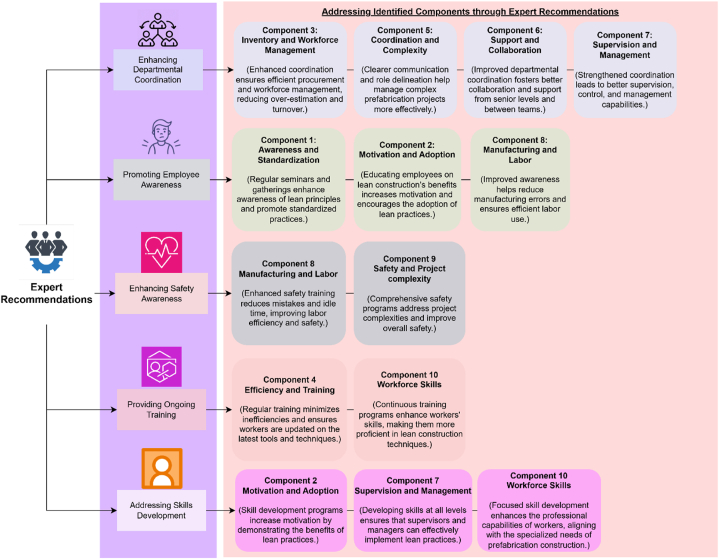
Chart displaying the alignment of expert recommendations and addressed components.

Fig. 5 depicts how each expert recommendation aligns with specific identified components, providing a detailed account of their approach to addressing each component.

## 5 Discussions

The findings of this research provide valuable insights into the barriers to the implementation of lean construction principles in the Indian prefabrication industry. As the analysis of the data shows, several key factors influence the implementation of lean construction and the barriers that need to be addressed to ensure its successful implementation. This section discusses the implications of these findings and their significance in the context of the Indian construction industry. The research identified a total of twenty-five barriers that hinder the implementation of lean construction in the Indian prefabrication sector. These barriers were uncovered through a comprehensive literature review, providing a robust foundation for understanding the challenges faced by industry professionals.

To make sense of these numerous barriers, the research employed exploratory factor analysis, resulting in the extraction of ten common components. These components represent clusters of related barriers and provide a more structured framework for addressing the challenges to lean construction implementation.

a) **Understanding and Standardization:** This component highlights the importance of raising understanding about lean construction and addressing issues related to the standardization of prefabrication processes and cost management [27,75].
b) **Motivation and Adoption:** Barriers related to motivation, competition, and resistance to change are grouped in this component, emphasizing the need for incentives and a willingness to adopt new tools and techniques [90].
c) **Inventory and Workforce Management:** Managing inventory and addressing workforce turnover are crucial factors within this component, indicating the significance of efficient material procurement and retaining skilled workers [91].
d) **Efficiency and Training:** Efficiency-related barriers, including waste due to double handling and the need for adequate training, are encompassed here. This emphasizes the importance of streamlining processes and enhancing workforce skills [25].
e) **Coordination and Complexity:** This component underscores the need for improved coordination among different departments, effective program planning, and managing the complexity of prefabrication works [92].
f) **Support and Collaboration:** Barriers related to support from senior levels, collaboration between teams, and efficient supply chain management are combined, emphasizing the need for a supportive organizational structure [93].
g) **Supervision and Management:** Issues related to supervision, coordination outside the construction department, and professional management capabilities are addressed within this component, underscoring the need for effective leadership [25].
h) **Manufacturing and Labor:** The component highlights the need to address issues such as manufacturing mistakes and labor inefficiencies, reducing waste and enhancing productivity [94].
i) **Safety and Project Complexity:** This component focuses on ensuring proper safety training and addressing the challenges presented by complex projects and a highly uncertain environment [95].
j) **Workforce Skills:** Finally, the component underscores the importance of having skilled workers to effectively implement lean construction practices [96].

The common components extracted from the barriers present several implications and avenues for future research and industry practice. Firstly, they offer a precise identification of critical barriers within the Indian prefabrication sector, aiding professionals in targeting their efforts effectively. Secondly, each component represents a cluster of related barriers, enabling the development of targeted solutions for more efficient implementation of lean construction. Moreover, the findings provide practical insights into overcoming barriers, facilitating easier implementation for industry professionals and policymakers. Additionally, understanding the interconnectedness of multiple barriers emphasizes the need for holistic approaches in addressing challenges within the prefabrication sector.

Subsequent to this, a discussion of the expert recommendations is presented.

a) Enhancing Departmental Coordination:

Effective coordination among departments is crucial for streamlining operations in prefabrication construction. By clearly defining roles and responsibilities, promoting transparent communication, and fostering an understanding of each department's challenges and operations, errors can be minimized and deadlines more consistently met. This approach not only enhances efficiency but also improves overall project management and delivery timelines. Implementing stringent adherence to deadlines and establishing clear lines of communication are essential steps in overcoming barriers related to departmental coordination.

b) Promoting Employee Understanding:

Regular employee gatherings and seminars focused on Lean Construction principles can significantly enhance understanding among workers in prefabrication firms. Educating employees about new techniques and procedures helps them understand their impact on project outcomes, fostering a culture of continuous improvement. Integrating Lean Construction terminology into contracts reinforces these principles and encourages compliance across all levels of the organization. This proactive approach ensures that Lean principles are ingrained in everyday practices, contributing to sustained improvements in project efficiency and quality.

c) Enhancing Safety:

Safety is paramount in prefabrication construction, where workers must be well-versed in handling machinery, tools, and equipment. Conducting comprehensive training sessions that cover safe handling practices, emergency procedures, and first aid protocols is essential for fostering a safety-conscious workforce. Regular post-training evaluations ensure that acquired knowledge is applied effectively on-site, while ongoing feedback mechanisms allow for continuous refinement of safety measures. By prioritizing safety understanding and training, prefabrication firms can mitigate risks, enhance worker well-being, and improve overall project safety outcomes.

d) Providing Ongoing Training:

Continuous training is essential for keeping workers abreast of new tools, equipment, and construction practices in prefabrication. Engaging knowledgeable instructors to educate workers on contemporary techniques not only enhances their skills but also empowers them to contribute more effectively to project efficiency and resource management. This ongoing training approach supports a culture of skill development and innovation within prefabrication firms, ensuring that workers remain competitive and adaptable in the evolving construction industry landscape.

e) Addressing Skills Development:

Skill development is critical in prefabrication construction, where specific expertise is required for efficient project execution. Lean construction education and training programs play a vital role in enhancing professional capabilities among workers. Conducting post-training evaluations helps assess individual skills and informs decisions related to career progression and compensation. By investing in skills development initiatives, prefabrication firms can cultivate a skilled workforce capable of meeting the industry's evolving demands, ultimately driving long-term business success and project excellence.

Furthermore, future studies can build upon these components, exploring their interrelationships and providing an ongoing research agenda for scholars interested in lean construction implementation in India. Moreover, implementing lean construction practices can advance the construction industry by promoting sustainability, efficiency, and competitiveness. Resolving the identified barriers aligns with sustainability and efficiency goals, contributing to environmentally friendly practices and resource optimization, thereby furthering the broader sustainability objectives of the construction sector.

This research significantly contributes to the understanding of lean construction implementation in the Indian prefabrication industry. The extracted common components offer a structured framework for addressing the barriers, providing valuable insights for industry professionals and policymakers. Moreover, these findings encourage further research and initiatives aimed at advancing the construction industry and promoting sustainability and efficiency.

## 6 Conclusions & recommendations

This research employed a comprehensive methodology to address challenges in implementing lean construction in prefabrication. It comprises three key steps: identifying barriers through a literature review, gathering insights from experts through questionnaires, and analyzing data using exploratory factor analysis. Literature analysis revealed 26 barriers, with “Inaccurate documentation” removed during pre-exploratory factor analysis. The top five barriers include a lack of coordination, insufficient awareness, inadequate safety training, limited training opportunities for new workers, and a lack of skilled workers. Additionally, ten common components summarize the findings. The study makes theoretical contributions by enhancing knowledge of challenges in the Indian prefabrication sector and advancing the theoretical framework for lean construction. Practically, it equips professionals to devise strategies and provides policymakers with a comprehensive view. It lays the foundation for future studies and encourages the implementation of lean construction in the Indian industry, contributing to sustainability. The study conducts an in-depth analysis of the top five barriers.

Below are the conclusions drawn from the prioritized recommendations aimed at enhancing lean construction implementation in the prefabrication sector.

• Clear role delineation, transparent communication, and understanding of operational challenges across departments are crucial for enhancing departmental coordination.
• Regular seminars and integration of Lean Construction principles into contracts can significantly improve worker proficiency and project outcomes.
• Comprehensive training on equipment handling, emergency protocols, and ongoing evaluations are essential for refining safety measures in prefabrication construction.
• Continuous education on modern practices and tools supports project efficiency and resource management.
• Investing in lean construction education enhances worker skills, readiness for industry demands, and supports career advancement opportunities.

While this study aims to shed light on lean construction barriers in India's prefabrication sector, it's important to recognize certain limitations. The focus on India may limit the applicability of findings to regions with different contexts, and while the methodology provided valuable insights, it may not fully capture the complexity of the issues. Purposive sampling aimed to include knowledgeable participants but may introduce bias due to researcher judgment, potentially limiting generalizability. Additionally, while the response rate was satisfactory, non-response bias could affect broader industry perspectives. The study's geographical focus may restrict applicability to other contexts, as different regions may face unique challenges, and self-reported data reliance may bias findings based on individual perceptions. Furthermore, industry practices are evolving, suggesting identified barriers could change over time with emerging technologies and methodologies.

The research findings and recommendations will serve as a useful resource and direction for the prefabricated sector to overcome the barriers causing hindrances in the implementation of lean construction. This research will increase lean construction utilization for prefabricated construction projects and the potentiality of the construction organization.

## Ethics declarations

Review and/or approval by an ethics committee was not needed for this study because this manuscript does not include human or animal participation.

## Funding statement:

This research received no external funding. The 10.13039/501100006333APC will be funded by 10.13039/100031019Digital Transformation Portfolio, 10.13039/501100007782Tshwane University of Technology, Staatsartillerie Rd, Pretoria West, Pretoria 0183, South Africa.

## Data availability statement

Data will be made available on request.

## CRediT authorship contribution statement

**Prafful Negi:** Writing – review & editing, Writing – original draft, Investigation, Data curation, Conceptualization. **Gaurav Thakur:** Writing – review & editing, Writing – original draft, Methodology, Data curation, Conceptualization. **Rajesh Singh:** Writing – review & editing, Writing – original draft, Methodology, Investigation, Data curation, Conceptualization. **Anita Gehlot:** Writing – review & editing, Writing – original draft, Methodology, Investigation, Data curation, Conceptualization. **Amit Kumar Thakur:** Writing – review & editing, Writing – original draft, Validation, Supervision, Investigation, Conceptualization. **Lovi Raj Gupta:** Writing – review & editing, Writing – original draft, Visualization, Validation, Supervision, Conceptualization. **Neeraj Priyadarshi:** Writing – review & editing, Writing – original draft, Validation, Supervision, Methodology. **Bhekisipho Twala:** Writing – review & editing, Writing – original draft, Validation, Supervision, Investigation, Funding acquisition.

## Declaration of competing interest

The authors declare that they have no known competing financial interests or personal relationships that could have appeared to influence the work reported in this paper.
