# Supplementary material for: Perception of lean construction implementation barriers in the indian prefabrication sector
Source: Heliyon. 2024 Aug 18;10(16):e36458. doi: 10.1016/j.heliyon.2024.e36458 (PMC11382055; doi:10.1016/j.heliyon.2024.e36458)
Supplement: Multimedia component 1 [file mmc1.docx]

**Questionnaire**

**(Lean Construction Implementation in Prefabrication Building)**

**Please indicate to what extent you agree to the following statements regarding factors hindering on-site lean construction of prefabricated buildings.**

**NOTE:** Lean construction is an adaptation of a manufacturing model that seeks to leverage careful operational research and practical design development to improve the project production process. In practice, this results in enhanced worker safety, timelier project completion, a stronger bottom line, and less waste for contractors in the construction space.

| **S. No.** | **Factors** | **Strongly Agree** | **Agree** | **Uncertain** | **Disagree** | **Strongly Disagree** |
| --- | --- | --- | --- | --- | --- | --- |
|  | Awareness about lean construction |  |  |  |  |  |
|  | Resistance to adopt better tools & techniques |  |  |  |  |  |
|  | Lack of motivation due to absence of relevant incentives |  |  |  |  |  |
|  | Unskilled workers |  |  |  |  |  |
|  | Insufficient program planning (ineffective scheduling and sequencing of materials, equipment, and labour) |  |  |  |  |  |
|  | Lack of coordination among different departments |  |  |  |  |  |
|  | Difficulty in collaboration between teams due to multilayer subcontracting |  |  |  |  |  |
|  | Market competition makes it difficult to adopt lean construction practices |  |  |  |  |  |
|  | Inadequate professional management capabilities of managers |  |  |  |  |  |
|  | Lack of effective supervision and control |  |  |  |  |  |
|  | High turnover of workforce |  |  |  |  |  |
|  | Lack of support from persons at senior levels |  |  |  |  |  |
|  | Insufficient standardization of prefabrication processes |  |  |  |  |  |
|  | Poor quality safety training |  |  |  |  |  |
|  | Insufficient fund and excessive cost saving during construction |  |  |  |  |  |
|  | Lack of coordination outside the construction department |  |  |  |  |  |
|  | Complex projects and highly uncertain environment |  |  |  |  |  |
|  | Waste due to double handling, over-communication, re-communication, and additional quality assurance checks/inspection |  |  |  |  |  |
|  | Limited training opportunities to new workers about tools, equipment and techniques |  |  |  |  |  |
|  | Construction workers are left idle on a site, leading to inflated labour expenditure |  |  |  |  |  |
|  | Mistake during manufacturing of prefabricated components |  |  |  |  |  |
|  | Complexity of prefabrication works |  |  |  |  |  |
|  | Inefficient supply chain management |  |  |  |  |  |
|  | Inaccurate documentation |  |  |  |  |  |
|  | Wasted talent due to excessive firm organizational hierarchies |  |  |  |  |  |
|  | Over-estimating, over-ordering, or the untimely procurement of inventory |  |  |  |  |  |

Name:

Designation:

Experience:
